# Maternal attitudes and home-based interventions for enhancing social, attention, and language skills in children with autism in Diwaniyah, Iraq

**DOI:** 10.3389/fpsyt.2025.1700859

**Published:** 2025-12-17

**Authors:** Aqeel Abd Al-Hamza Marhoon, Khamees Bandar Obaid

**Affiliations:** Department of Pediatric Nursing, College of Nursing, University of Babylon, Babylon, Iraq

**Keywords:** autism spectrum disorder, home-based interventions, Iraq, maternal attitudes, social skills development

## Abstract

**Background:**

Autism Spectrum Disorder (ASD) presents significant developmental challenges, particularly in low-resource settings. Maternal attitudes and engagement in home-based interventions are critical for supporting children’s social, attention, and language development.

**Aims:**

To assess maternal attitudes toward children with ASD and evaluate the implementation of home-based interventions targeting social, attention, and language skills in Diwaniyah, Iraq.

**Methods:**

A descriptive, cross-sectional study was conducted between April and August 2025, involving a census sample of 205 mothers of children with ASD from three autism centers in Diwaniyah. Data were collected through structured face-to-face interviews using a validated questionnaire. Statistical analyses were performed using SPSS version 25, with descriptive statistics and Chi-square tests applied to examine the data.

**Results:**

The majority of mothers (79.0%) exhibited positive attitudes toward their children with ASD. Positive maternal attitudes were significantly associated with higher education, urban residence, sufficient income, attendance at educational sessions, and absence of family mental illness (p < 0.05). Home-based intervention implementation was high in most domains: 67.3% for social skills, 88.8% for attention, and 67.8% for language. A statistically significant association was found between positive maternal attitudes and the use of home-based interventions targeting social skills (P value = 0.001), but not for attention or language interventions (P values > 0.05). After adjusting for potential confounders, the results show that maternal attitudes were significantly associated only with the likelihood of using home-based interventions within the social domain (adjusted odds ratio = 1.021; 95% CI: 0.208-5.010; p = 0.001).

**Conclusion:**

Maternal attitudes significantly influence the implementation of home-based social skill interventions in children with ASD. Strengthening caregiver training and psychosocial support, particularly in underserved areas, is essential to enhance home-based developmental outcomes in low-resource contexts like Diwaniyah.

## Introduction

Autism Spectrum Disorder (ASD) is a complex neurodevelopmental disorder characterized by persistent deficits in social interaction and communication, as well as restricted, repetitive behaviors and interests (1, 2). Symptoms usually appear in early childhood and can significantly impair social, academic, and daily functioning across the lifespan (2). Globally, ASD affects approximately 28 million individuals, including about 3 million children under the age of five, reflecting its widespread public health burden (3). As the prevalence continues to rise, addressing ASD requires not only clinical approaches but also integrated family and community-based strategies (4).

Mothers are typically the primary caregivers for children with ASD and play a critical role in managing the child’s needs. Their responsibilities often include daily caregiving, behavior regulation, facilitating learning, and emotional support (5, 6). Maternal attitudes encompassing perceptions, beliefs, and emotional responses toward the child’s condition, can significantly shape the caregiving environment and influence the child’s developmental outcomes (7). Research shows that positive maternal attitudes contribute to improved communication, better social skills, and higher adherence to therapeutic interventions (8). On the other hand, negative attitudes or elevated stress levels may reduce maternal engagement and exacerbate behavioral challenges in the child (9).

Multiple factors contribute to shaping maternal attitudes, including maternal education, income level, family size, cultural background, and the presence of mental illness in the family (10). In low- and middle-income countries (LMICs), such as Iraq, these challenges are compounded by limited access to autism-related services, minimal caregiver training, and high levels of stigma, particularly in rural and underserved communities. These limitations not only impact the child’s development but also increase caregiver burden and hinder early intervention efforts.

Home-based interventions defined as parent-implemented strategies within the home setting, have gained international attention for their accessibility and cost-effectiveness, particularly in LMICs. These approaches focus on enhancing social interaction, attention, and communication through daily routines, structured play, visual supports, and imitation tasks (11, 12). Studies have shown that consistent use of home-based strategies can lead to significant improvements in joint attention, expressive language, and adaptive behaviors in children with ASD (13, 14). Moreover, such interventions empower mothers to become active facilitators of their child’s developmental progress and promote more sustainable care models in resource-limited settings. However, the effectiveness of home-based interventions is closely tied to maternal engagement, knowledge, and attitude toward the intervention process. Therefore, understanding maternal perceptions and the degree to which they apply developmental strategies at home is crucial for the success of these interventions (15). Research also suggests that culturally tailored caregiver training improves strategy implementation, reduces stress, and enhances parenting self-efficacy (13, 15).

In Iraq, and specifically in the city of Diwaniyah, research on ASD remains scarce, and no comprehensive studies have examined the relationship between maternal attitudes and the use of home-based interventions. Diwaniyah, located in central Iraq, faces systemic challenges in both healthcare and education systems, which restrict access to early ASD diagnosis and treatment. Specialized autism centers are few, and families in rural or low-income settings are especially vulnerable. Social stigma, limited awareness, and economic hardship further complicate caregiving responsibilities. In addition, the high prevalence of consanguineous marriage in Iraq has been associated with increased risks of neurodevelopmental conditions, including ASD (16, 17). Despite these challenges, mothers in Diwaniyah play a vital role in the daily care and developmental support of children with ASD. Yet, little is known about their beliefs, emotional responses, or the specific home-based strategies they employ. Most existing research in the region focuses on either clinical outcomes or generalized parental stress, without integrating maternal attitude assessments with actual caregiving behaviors. The absence of such data limits the design of effective, culturally appropriate intervention programs.

This study addresses this critical gap by exploring maternal attitudes and their association with the implementation of home-based interventions targeting social, attention, and language skills in children with ASD. The focus on Diwaniyah allows for a context-specific analysis of caregiving within a low-resource environment. The findings are expected to contribute to the development of tailored support systems that empower mothers, improve child outcomes, and inform broader policy and practice in Iraq and similar LMICs. Therefore, the main aim of this study is to assess maternal attitudes and evaluate the implementation of home-based interventions that target social, attention, and language skills in children with ASD in Diwaniyah, Iraq.

## Materials and methods

### Study design

This descriptive, cross-sectional study was conducted over a five-month period, from April to August 2025, in order to assess maternal attitudes and the use of home-based interventions aimed at enhancing social, attention, and language skills in children diagnosed with ASD. The design was chosen to provide a snapshot of current practices and perceptions among mothers of children with ASD in Diwaniyah, Iraq.

### Study setting

The study was carried out in Diwaniyah, a governorate in central-southern Iraq. Data collection took place in three major centers that specialize in autism care and education: Raja Governmental Institute, Ruqaya Private Institute for ASD, and the Al-Sabatin Foundation for Autism and Growth Forests, which is affiliated with the Health and Medical Education Authority. These centers serve as key referral and treatment facilities for both urban and rural populations and offer diagnostic, therapeutic, and educational services for children with ASD.

### Inclusion and exclusion criteria

Participants in this study were mothers of children diagnosed with ASD aged between 2 and 16 years, residing in either urban or rural areas of Diwaniyah governorate. To be eligible, the child had to be officially registered in one of the three participating autism centers. Additionally, mothers had to express willingness to participate voluntarily and provide written informed consent. Mothers were excluded if their children had comorbid severe intellectual disabilities or physical impairments that could hinder participation in home-based interventions. Incomplete responses and those who were unwilling or unable to provide informed consent were also excluded from the study.

### Sample size and sampling strategy

A census sampling method was employed for this study. All mothers of children diagnosed with ASD who met the inclusion criteria and were registered at the three selected autism centers in Diwaniyah during the study period (April to August 2025) were included. The total number of eligible participants identified was 205, and all were invited to participate. This comprehensive inclusion ensured that the entire accessible population of mothers within the specified centers was represented, enhancing the study’s generalizability within the local context.

### Data collection

Data were collected using a structured, interview-based questionnaire, divided into three main sections. The first section gathered demographic and socioeconomic information, including maternal age, education level, place of residence, household income, family size, marital status, presence of consanguineous marriage, and previous participation in autism-related training or awareness programs. The second section evaluated maternal attitudes toward children with ASD using a set of Likert-scale items that captured cognitive perceptions, emotional reactions, and behavioral inclinations regarding the child’s condition. This section was adapted from a previously validated tool in culturally comparable populations (18); maternal attitudes toward children with ASD, comprising 15 items assessing caregiving practices, affection, discipline, and communication, scored on a 3-point Likert scale (1 = Disagree, 2 = Neutral, 3 = Agree), with higher scores reflecting more positive attitudes. The third section examined the implementation of home-based intervention strategies by mothers, focusing on their involvement in structured activities aimed at enhancing their child’s social interaction, attention span, and language skills. The items in this section were derived from established frameworks and prior studies in the field (19, 20); mother-led home-based interventions, encompassing practices to enhance social, attention, and language communication skills, with each domain including 7–9 items rated on a 3-point frequency scale (1 = Never, 2 = Sometimes, 3 = Always). The overall attitude and intervention scores were categorized as low, moderate, or high based on mean score cut-offs. To ensure content validity, the questionnaire was reviewed by a panel of six subject-matter experts in pediatric nursing, child mental health, and autism therapy. The instrument was translated into Arabic and underwent a forward and backward translation process to maintain linguistic and conceptual equivalence.

Additionally, eligible participants were approached during scheduled visits to the autism centers. Trained researchers conducted face-to-face interviews using the structured questionnaire in a private and quiet setting to ensure participant comfort and confidentiality. The purpose and procedures of the study were clearly explained before each interview. Participants were given sufficient time to respond to each item, and clarification was provided as needed to ensure accurate and complete responses.

### Pilot study

A pilot study was conducted with 20 mothers to assess the clarity, cultural appropriateness, and feasibility of the data collection tool. Based on participant feedback, minor revisions were made to enhance the language and structure of the questionnaire. Reliability analysis using Cronbach’s alpha demonstrated strong internal consistency, with values of 0.87 for the maternal attitudes section and 0.83 for the home-based intervention practices section. These results confirm the reliability and suitability of the instrument for use in the target population. Data from the pilot study were excluded from the final analysis.

### Statistical analysis

Data analysis was performed using SPSS program, version 25. Descriptive statistics, including frequencies, percentages, means, and standard deviations, were used to summarize demographic data and responses to questionnaire items. To determine the relationships between demographic characteristics, maternal attitudes, and the implementation of home-based interventions, Chi-square test was used. A p-value less than 0.05 was considered statistically significant.

### Ethical approval

Ethical approval for the study was obtained from the Scientific Research Ethics Committee at the University of Babylon. Written informed consent was secured from all participants after explaining the study’s purpose, procedures, and voluntary nature. Participants were assured of their right to withdraw at any stage without any consequences. All responses were treated with strict confidentiality, and data were stored securely to prevent unauthorized access. The study was conducted with full respect for the participants’ personal beliefs, cultural norms, and emotional well-being.

## Results

A total of 205 mothers of children with ASD participated in the current study and were included in the final analysis. The findings indicated that approximately half of the mothers, 100 (48.8%), were between the ages of 29 and 38 years. In terms of education, 55 (26.7%) held a bachelor’s degree, while 9 (4.4%) had a postgraduate degree. The majority of participants, 187 (91.2%), resided in urban areas. Additionally, 119 mothers (58.0%) reported having families with 3 to 5 members, and 56 (27.3%) indicated that their monthly household income was insufficient. Regarding health and family background, 28 mothers (13.7%) reported a family history of psychiatric or mental health issues. Consanguineous marriage between the parents was reported by 78 (38.0%) of the participants, and 29 (14.1%) stated they had attended educational sessions on how to manage and support their child with ASD. These details are summarized in **Table 1**.

**Table 1:** Characteristics of the study participants.

Variables	Frequency (n=205)	Percentage (100%)
Mother age		
18–28 years	24	11.7
29–38 years	100	48.8
39 years and more	81	39.5
Mother level of education		
Cannot read and write	8	3.9
Reads and writes	20	9.8
Elementary	12	5.9
Secondary	58	28.3
Diploma	43	21.0
Bachelor	55	26.7
Postgraduate	9	4.4
Residences of family		
Rural	18	8.8
Urban	187	91.2
Number of family member		
3 – 5	119	58.0
6 – 9	50	24.4
10 and more	36	17.6
Family monthly incomes		
Not enough	56	27.3
Somewhat enough	70	34.1
Enough	79	38.6
Psychiatric and mantel history of family		
No	177	86.3
Yes	28	13.7
Consanguineous marriage between mother and father		
No	127	62.0
Yes	78	38.0
Have you attended educational sessions on how to deal with your child with autism spectrum disorder?		
No	176	85.9
Yes	29	14.1
Characteristics of the child with autism spectrum disorder		
Child gender		
Male	133	64.9
Female	72	35.1
Child age		
2–7 years	129	62.9
8–13 years	61	29.8
14–16 years	15	7.3
The order of the child with autism spectrum disorder in the family		
Lonely	8	3.9
First	78	38.0
Second	26	12.7
Third or more	93	45.4
Severity of autism spectrum disorder		
Mild	52	25.4
Moderate	124	60.5
Severe	29	14.1
Duration of diagnosis		
1–3 years	66	32.2
4–6 years	68	33.2
7 years and more	71	34.6

**Table 1** also presents characteristics of the children with ASD. The results showed that 133 (64.9%) of the children were male, and 72 (35.1%) were female. A significant proportion, 129 children (62.9%), were between 2 and 7 years old. With respect to birth order, 78 children (38.0%) were the first-born in their families. In terms of ASD severity, 52 children (25.4%) were reported to have mild symptoms, 124 (60.5%) moderate, and 29 (14.1%) severe. Additionally, 68 children (33.2%) had been diagnosed with ASD for a duration of 4 to 6 years.

As shown in **Table 2**, the majority of mothers demonstrated positive and supportive parenting behaviors toward their children with ASD. Specifically, 77.1% reported that they let their child rest or carry them when tired during outings, and 87.3% expressed affection through hugging and speaking gently. About 67.4% of mothers responded to their child’s frustration by asking what was wrong, while 59.0% rejected the idea of postponing playtime even when busy. In terms of discipline and interaction, 46.4% of mothers discussed misbehavior with their child to encourage better choices, and 57.1% reported spending time playing games at home. Regarding family outings, 46.8% considered their child’s preferences, and 56.1% disagreed with denying a toy during shopping if the child insisted. Additionally, 55.1% calmed their child during fussiness, and 51.7% used direct instructions when their child refused to comply. Further, 44.4% addressed conflicts between children by having their child return toys taken from peers. Most mothers (76.6%) disagreed with leaving their child alone at bedtime if still playing, and 85.9% did not tolerate the use of inappropriate language such as “stupid” or “idiot.” In public spaces, 65.3% ensured their child remained quiet, and 52.7% retaught the importance of keeping promises when broken.

**Table 2:** Mothers’ attitudes for children with autism spectrum disorder.

Variables	Frequency (n=205)	Percentage (100%)	Mean ± SD
1. When you go somewhere and feel that your child is tired, you take a rest or hold him/her.			
Disagree	14	6.8	2.70 ± 0.58
Neutral	33	16.1	
Agree	158	77.1	
2. You show affection by hugging your child and speaking in a tender tone.			
Disagree	14	6.8	2.80 ± 0.54
Neutral	12	5.9	
Agree	179	87.3	
3. When you think your child is frustrated, you ask, ‘What’s wrong?’			
Disagree	38	18.5	2.49 ± 0.79
Neutral	29	14.1	
Agree	138	67.4	
4. When you are busy, you put off playing with him/her even if your child wants to play with you.			
Disagree	121	59.0	1.61 ± 0.80
Neutral	42	20.5	
Agree	42	20.5	
5. When your child does something wrong, you ask him/her why he/she did it and discuss how he/she could have done better.			
Disagree	56	27.3	2.19 ± 0.83
Neutral	54	26.3	
Agree	95	46.4	
6. When you are at home, you spend time playing ball or games with your child.			
Disagree	25	12.2	2.45 ± 0.70
Neutral	63	30.7	
Agree	117	57.1	
7. When going out with your family, you consider where your child wants to go, as much as possible, not just for the convenience of the parents.			
Disagree	25	12.2	2.35 ± 0.68
Neutral	84	41.0	
Agree	96	46.8	
8. When you go shopping and don’t plan to buy a toy, even if your child says he/she wants a toy and doesn’t move from the sales floor, you don’t buy the toy.			
Disagree	115	56.1	1.58 ± 0.72
Neutral	61	29.8	
Agree	29	14.1	
9. When your child is making a fuss about what he is doing, you keep him/her quiet.			
Disagree	50	24.4	2.31 ± 0.83
Neutral	42	20.5	
Agree	113	55.1	
10. When your child does not do what he/she is supposed to do, you say ‘do it’.			
Disagree	53	25.9	2.26 ± 0.84
Neutral	46	22.4	
Agree	106	51.7	
11. When your child is playing with a friend and forcibly takes away a toy that the friend is using, you have your child return it.			
Disagree	75	36.6	2.08 ± 0.89
Neutral	39	19.0	
Agree	91	44.4	
12. When it’s time for your child to go to bed, you leave him/her alone when he/she is playing and not sleeping.			
Disagree	157	76.6	1.38 ± 0.72
Neutral	19	9.3	
Agree	29	14.1	
13. You don’t care if your child uses bad language towards you (‘stupid’, ‘idiot’, etc).			
Disagree	176	85.9	1.21 ± 0.56
Neutral	14	6.8	
Agree	15	7.3	
14. You keep your child quiet in places that must be quiet, such as libraries and movie theatres.			
Disagree	45	22.0	2.43 ± 0.82
Neutral	26	12.7	
Agree	134	65.3	
15. When your child doesn’t keep a promise with you, you teach that promise again.			
Disagree	41	20.0	2.33 ± 0.78
Neutral	56	27.3	
Agree	108	52.7	
Overall assessment of mothers’ attitudes			
Negative attitudes (≤ 2.00)	43	21.0	2.14 ± 0.35
Positive attitudes (> 2.00)	162	79.0	

Overall, the assessment of mothers’ attitudes revealed that 162 (79.0%) had positive attitudes toward children with ASD, while only 43 (21.0%) displayed negative attitudes, as illustrated in **Table 2**.

**Table 3** summarizes maternal home-based interventions targeting social skill development in children with ASD. The majority of mothers actively encouraged peer interactions, with 71.2% promoting group play and friend selection, and 58.6% assigning their child roles in family activities. Social communication training was also common, as 70.7% taught social gestures and 53.6% emphasized eye contact. However, only 36.1% regularly involved their child in clubs or nurseries, while 57.6% occasionally encouraged peer observation and imitation. Attendance at social events varied, with 38.0% consistently accompanying their child to birthdays, whereas 44.4% never engaged in outings such as trips or summer camps. Nearly half (47.3%) fostered participation in preferred group activities. Overall, maternal interventions were rated as high by 67.3%, moderate by 28.3%, and low by 4.4% of mothers.

**Table 3:** Mother home-based interventions for enhancing social, attention, and language skills in children with autism spectrum disorder.

Variables	Frequency (n=205)	Percentage (100%)	Mean ± SD
Mother home-based interventions for enhancing social skills			
1. Encourage the child to establish relationships with other children through group play and the free choice of friends.			
Never	6	2.9	2.68 ± 0.52
Sometimes	53	25.9	
Always	146	71.2	
2. Assign the child a role in family participation.			
Never	13	6.3	2.52 ± 0.61
Sometimes	72	35.1	
Always	120	58.6	
3. Train the child in social gestures such as hand gesturing (bai) and head movement to express rejection or approval.			
Never	9	4.4	2.66 ± 0.55
Sometimes	51	24.9	
Always	145	70.7	
4. Training the child to respond to meeting eyes when talking to others.			
Never	12	5.9	2.48 ± 0.60
Sometimes	83	40.5	
Always	110	53.6	
5. Involving the child in one of the clubs or nurseries.			
Never	73	35.6	2.00 ± 0.84
Sometimes	58	28.3	
Always	74	36.1	
6. I encourage the child to focus his/her observation and imitate other children.			
Never	17	8.3	2.26 ± 0.59
Sometimes	118	57.6	
Always	70	34.1	
7. I accompany the child on birthdays and similar occasions.			
Never	51	24.9	2.13 ± 0.78
Sometimes	76	37.1	
Always	78	38.0	
8. I take the child on trips or summer camps.			
Never	91	44.4	1.72 ± 0.73
Sometimes	80	39.0	
Always	34	16.6	
9. Encourage the child to share with children in group actions he loves.			
Never	47	22.9	2.24 ± 0.80
Sometimes	61	29.8	
Always	97	47.3	
Overall assessment of mother home-based interventions for enhancing social skills in children with autism spectrum disorder.			
Low (1-1.66)	9	4.4	2.30 ± 0.40
Moderate (1.67-2.33)	58	28.3	
High (> 2.33)	138	67.3	
Mother home-based interventions for enhancing attention and focus skills			
1. I reached the child’s gaze for a certain thing for a long time.			
Never	28	13.7	2.36 ± 0.71
Sometimes	75	36.6	
Always	102	49.7	
2. I reward the child if he responds with me.			
Never	19	9.3	2.58 ± 0.65
Sometimes	49	23.9	
Always	137	66.8	
3. I give the child simple and clear instructions when I am training a specific skill.			
Never	14	6.8	2.61 ± 0.61
Sometimes	52	25.4	
Always	139	67.8	
4. I set dates for food and play for the child.			
Never	13	6.3	2.58 ± 0.61
Sometimes	60	29.3	
Always	132	64.4	
5. I encourage the child to play some games and activities that help focus, such as: Matching photos, coloring pictures, and building blocks.			
Never	14	6.8	2.60 ± 0.61
Sometimes	54	26.3	
Always	137	66.9	
6. Constantly grabbing the child’s attention, such as fumbling and calling the child with a smile.			
Never	30	14.6	2.55 ± 0.73
Sometimes	33	16.1	
Always	142	69.3	
Overall assessment of mother home-based interventions for enhancing attention and focus skills in children with autism spectrum disorder.			
Low (1-1.66)	10	4.9	2.54 ± 0.41
Moderate (1.67-2.33)	13	6.3	
High (> 2.33)	182	88.8	
Mother home-based interventions for enhancing language communication skills			
1. Use one word or short sentences when talking to the child.			
Never	15	7.3	2.58 ± 0.62
Sometimes	57	27.8	
Always	133	64.9	
2. Use a picture or a picture referred to when talking to the child, to help train a specific skill.			
Never	47	22.9	2.08 ± 0.73
Sometimes	94	45.9	
Always	64	31.2	
3. I avoid using questions that need to be explained with the child.			
Never	41	20.0	2.20 ± 0.75
Sometimes	81	39.5	
Always	83	40.5	
4. Use the signals that get the child’s attention when communicating with him/her.			
Never	36	17.6	2.51 ± 0.77
Sometimes	29	14.1	
Always	140	68.3	
5. Encourage the child to pronounce the word for the things being referred to.			
Never	13	6.3	2.80 ± 0.53
Sometimes	15	7.3	
Always	177	86.4	
6. Use pictures of everyday objects to facilitate communication with the child; encourage the child to go through an activity that develops control over his/her mouth muscles, such as: Blowing bubbles, blow out a candle.			
Never	40	19.5	2.31 ± 0.78
Sometimes	61	29.8	
Always	104	50.7	
7. Train the child in the skill of imitation, “a kinetic mimicry, such as: Tongue movement.			
Never	18	8.8	2.54 ± 0.65
Sometimes	58	28.3	
Always	129	62.9	
Overall assessment of mother home-based interventions for enhancing language communication skills in children with autism spectrum disorder.			
Low (1-1.66)	14	6.8	2.42 ± 0.46
Moderate (1.67-2.33)	52	25.4	
High (> 2.33)	139	67.8	

Regarding maternal home-based interventions for enhancing attention and focus skills in children with ASD, the results indicated that 102 mothers (49.7%) consistently encouraged their child to maintain gaze on a specific object for an extended period. A majority, 137 (66.8%), reported rewarding their child when responding to their name, and 139 (67.8%) provided simple, clear instructions during skill training. Additionally, 132 mothers (64.4%) established regular schedules for food and play, while 137 (66.9%) encouraged engagement in activities designed to improve focus, such as matching photos, coloring, and building blocks. Moreover, 142 mothers (69.3%) actively captured their child’s attention through actions like fumbling and calling with a smile. Overall, the assessment of maternal interventions for attention and focus was rated as high by 88.8% of mothers, moderate by 6.3%, and low by 4.9%.

With regard to maternal home-based interventions for enhancing language and communication skills in children with ASD, the findings showed that 133 mothers (64.9%) consistently used single words or short sentences when speaking to their child, and 94 (45.9%) occasionally used pictures or visual references to support skill development. A total of 83 mothers (40.5%) regularly avoided using complex questions that required detailed explanations. Additionally, 140 (68.3%) reported always using signals to capture the child’s attention during communication, while 177 (86.4%) encouraged the child to verbalize the names of objects. Furthermore, 104 mothers (50.7%) used images of everyday items to support communication, and a majority encouraged participation in oral motor activities such as blowing bubbles or candles. Finally, 129 mothers (62.9%) consistently trained their child in imitation skills, including kinetic mimicry like tongue movements. Overall, the assessment of maternal interventions for language and communication development was classified as high in 139 cases (67.8%), moderate in 52 (25.4%), and low in 14 (6.8%).

**Table 4** presents the association between maternal attitudes and various sociodemographic and clinical characteristics of the study participants. The analysis revealed statistically significant relationships between maternal attitudes and the following variables: maternal level of education, place of residence, number of family members, monthly family income, family history of psychiatric or mental illness, consanguinity between parents, attendance at educational sessions on managing children with ASD, birth order of the child with ASD, severity of ASD, and duration since diagnosis. For all these variables, the associations with maternal attitudes were statistically significant, with p-values < 0.05.

**Table 4:** Association between maternal attitudes and characteristics of the study participants.

Variables	Attitudes categories		P value
	Negative attitudes N=43 (21.0%)	Positive attitudes N=162 (79.0%)	
Mother age			
18–28 years	8.0 (33.3)	16 (66.7)	0.237
29–38 years	21 (21.0)	79 (79.0)	
39 years and more	14 (17.3)	67 (82.7)	
Mother level of education			
Cannot read and write	0.0 (0.0)	8.0 (100.0)	**0.001**
Reads and writes	12 (60.0)	8.0 (40.0)	
Elementary	2.0 (16.7)	10 (83.3)	
Secondary	13 (22.4)	45 (77.6)	
Diploma	1.0 (2.3)	42 (97.7)	
Bachelor	15 (27.3)	40 (72.7)	
Postgraduate	0.0 (0.0)	9.0 (100.0)	
Residences of family			
Rural	0.0 (0.0)	18 (100.0)	**0.012**
Urban	43 (23.0)	144 (77.0)	
Number of family member			
3 – 5	30 (25.2)	89 (74.8)	**0.001**
6 – 9	0.0 (0.0)	50 (100.0)	
10 and more	13 (36.1)	23 (63.9)	
Family monthly incomes			
Not enough	12 (21.4)	44 (78.6)	**0.001**
Somewhat enough	28 (40.0)	42 (60.0)	
Enough	3.0 (3.8)	76 (96.2)	
Psychiatric and mantel history of family			
No	31 (17.5)	146 (82.5)	**0.004**
Yes	12 (42.9)	16 (57.1)	
Consanguineous marriage between mother and father			
No	42 (33.1)	85 (66.9)	**0.001**
Yes	1.0 (1.3)	77 (98.7)	
Have you attended educational sessions on how to deal with your child with autism spectrum disorder?			
No	43 (24.4)	133 (75.6)	**0.001**
Yes	0.0 (0.0)	29 (100.0)	
Characteristics of the child with autism spectrum disorder			
Child gender			
Male	28 (21.1)	105 (78.9)	0.561
Female	15 (20.8)	57 (79.2)	
Child age			
2–7 years	30 (23.3)	99 (76.7)	0.111
8–13 years	13 (21.3)	48 (78.7)	
14–16 years	0.0 (0.0)	15 (100.0)	
The order of the child with autism spectrum disorder in the family			
Lonely	0.0 (0.0)	8.0 (100.0)	**0.001**
First	28 (35.9)	50 (64.1)	
Second	1.0 (3.8)	25 (96.2)	
Third or more	14 (15.1)	79 (84.9)	
Severity of autism spectrum disorder			
Mild	0.0 (0.0)	52 (100.0)	**0.001**
Moderate	27 (21.8)	97 (78.2)	
Severe	16 (55.2)	13 (44.8)	
Duration of diagnosis			
1–3 years	12 (18.2)	54 (81.8)	**0.001**
4–6 years	6.0 (8.8)	62 (91.2)	
7 years and more	25 (35.2)	46 (64.8)	

Bold values denote statistical significance at p < 0.05.

**Table 5** illustrates the relationship between maternal attitudes and the implementation of home-based interventions aimed at improving social, attention, and language skills in children with ASD. The results indicated a statistically significant association between maternal attitude categories and the overall use of home-based interventions targeting social skills, with a p-value of 0.001. Conversely, no statistically significant associations were observed between maternal attitudes and the overall use of home-based interventions for enhancing attention and focus skills or language and communication abilities in children with ASD (p-values > 0.05 for both domains).

**Table 5:** Association between maternal attitudes and home-based interventions for enhancing social, attention, and language skills in children with autism.

Variables	Attitudes categories		Total N=205 (100%)	P value
	Negative attitudes N=43 (21.0%)	Positive attitudes N=162 (79.0%)		
Overall assessment of mother home-based interventions for enhancing social skills in children with autism spectrum disorder.				
Low (1-1.66)	2.0 (1.0)	7.0 (3.4)	9.0 (4.4)	**0.001**
Moderate (1.67-2.33)	27 (13.2)	31 (15.1)	58 (28.3)	
High (> 2.33)	14 (6.8)	124 (60.5)	138 (67.3)	
Overall assessment of mother home-based interventions for enhancing attention and focus skills in children with autism spectrum disorder.				
Low (1-1.66)	2.0 (1.0)	8.0 (3.9)	10 (4.9)	0.669
Moderate (1.67-2.33)	4.0 (1.9)	9.0 (4.4)	13 (6.3)	
High (> 2.33)	37 (18.1)	145 (70.7)	182 (88.8)	
Overall assessment of mother home-based interventions for enhancing language communication skills in children with autism spectrum disorder.				
Low (1-1.66)	3.0 (1.4)	11 (5.4)	14 (6.8)	0.125
Moderate (1.67-2.33)	16 (7.8)	36 (17.6)	52 (25.4)	
High (> 2.33)	24 (11.7)	115 (56.1)	139 (67.8)	

Bold values denote statistical significance at p < 0.05.

**Table 6** displays the adjusted odds ratios and 95% confidence intervals assessing the relationship between maternal attitudes and the use of home-based interventions aimed at improving social, attention, and language communication skills in children with autism. After controlling for potential confounders, the results show that maternal attitudes were significantly linked only to the use of home-based interventions in the social domain (adjusted odds ratio = 1.021; 95% CI: 0.208-5.010; p = 0.001). This finding indicates that strong maternal attitudes may play a meaningful role in promoting social skill development at home for children with autism.

**Table 6:** Adjusted odds ratios with 95% confidence intervals for maternal attitudes and home-based interventions in children with autism.

Variables	B (SE)	Adjusted odds ratio (95% CI)	Wald statistics	P value
Overall assessment of mother home-based interventions for enhancing social skills in children with autism spectrum disorder.				
Low		Ref		–
Moderate	1.115 (0.844)	3.048 (0.583 - 15.936)	1.745	0.187
High	0.020 (0.812)	1.021 (0.208 - 5.010)	0.980	0.001
Overall assessment of mother home-based interventions for enhancing attention and focus skills in children with autism spectrum disorder.				
Low		Ref		–
Moderate	0.575 (0.993)	1.778 (0.254 - 12.449)	0.336	0.562
High	-.928 (0.850)	0.395 (0.075 - 2.090)	1.193	0.275
Overall assessment of mother home-based interventions for enhancing language communication skills in children with autism spectrum disorder.				
Low		Ref		–
Moderate	0.488 (0.717)	1.630 (0.400 - 6.647)	0.464	0.496
High	-.268 (0.689)	0.765 (0.198 - 2.953)	0.151	0.698

Statistical testing using logistic regression. Ref, reference category; Adjusted odds ratio using 95% confidence interval in multinomial logistic regression analysis; CI, confidence interval; B, slop. Difference is significant at the 0.05 level (2-tailed).

## Discussion

This study examined maternal attitudes and the implementation of home-based interventions designed to enhance social, attention, and language skills in children with ASD within the context of Diwaniyah, Iraq. The findings provide important insights into the caregiving dynamics and intervention practices in a low-resource setting, where autism-related services are limited and sociocultural challenges are pronounced.

### Maternal attitudes toward children with ASD

The finding that 79.0% of mothers exhibited positive attitudes toward their children with ASD is a critical observation, as maternal perceptions fundamentally shape caregiving behavior and intervention success. This result aligns with research from diverse settings indicating that positive parental attitudes correlate with more adaptive caregiving approaches and better child developmental outcomes (8). For instance, the researchers demonstrated that favorable maternal attitudes enhance engagement with home-based therapies and improve social communication skills in children with ASD (19). In contrast, negative maternal attitudes are associated with increased caregiver stress and reduced intervention adherence (9). The relatively high prevalence of positive attitudes in this study suggests a resilient caregiving environment despite systemic barriers, which may be partly attributable to cultural factors emphasizing family cohesion and maternal responsibility.

Sociodemographic factors emerged as significant correlates of maternal attitudes. Mothers with higher educational attainment, urban residency, greater family income, and prior attendance at autism-related educational programs were more likely to demonstrate positive attitudes. These findings mirror those of prior investigations in LMICs and high-income countries alike, where maternal education and socioeconomic status consistently predict knowledge, perceptions, and caregiving efficacy (21). The observed associations highlight the critical role of caregiver empowerment through education and access to resources in shaping constructive attitudes. The relatively low rate of mothers attending educational sessions (14.1%) underscores a critical gap in service provision in Diwaniyah, indicating a need for expanded outreach and accessible caregiver training to further improve attitudes and outcomes.

Interestingly, the study identified a significant association between consanguineous marriage and maternal attitudes, a factor particularly relevant in the Iraqi context given the high prevalence of consanguinity and its association with increased neurodevelopmental risks including ASD (22). This finding may reflect heightened awareness or concern among mothers from consanguineous unions regarding their child’s developmental challenges, potentially influencing their attitudes either positively through increased vigilance or negatively due to stigma. This nuanced relationship warrants further exploration to tailor culturally sensitive intervention and counseling programs.

### Implementation of home-based interventions for children with ASD

The assessment of home-based interventions revealed robust maternal engagement, particularly in strategies targeting social and attention skills. A majority of mothers encouraged peer interaction through play and social gestures, incorporated structured routines and clear instructions to enhance attention, and used simplified speech and imitation exercises to promote language development. These practices are consistent with evidence-based recommendations emphasizing parent-mediated interventions as feasible, cost-effective, and impactful approaches in resource-limited settings (19, 23).

However, the frequency and consistency of these interventions varied across domains and participants. For example, only 36.1% of mothers regularly involved their child in external social settings such as clubs or nurseries, and less than half used visual supports to enhance communication. This partial implementation may reflect practical challenges such as limited access to inclusive social environments, caregiver time constraints, and low awareness of intervention techniques. These findings correspond with reports from similar LMIC contexts where infrastructural limitations, stigma, and financial hardship constrain comprehensive intervention delivery (24).

A noteworthy finding is the statistically significant relationship between maternal attitudes and home-based interventions targeting social skills but not attention or language skills. This suggests that positive maternal perceptions may directly motivate social engagement efforts, which are more observable and emotionally reinforcing to caregivers. In contrast, interventions for attention and language may require more specialized knowledge or sustained effort that mothers find more difficult to consistently implement without professional support. This differential association aligns with prior research indicating that social skill interventions are often prioritized by caregivers due to their immediate relevance to daily social functioning (25). In the current study, maternal attitudes were significantly linked to social-skill interventions but not attention or language activities, likely because social interactions provide immediate feedback and reinforcement, motivating caregiver engagement. In contrast, attention and language interventions are more structured and technical, requiring specialized knowledge or training. This suggests that while positive attitudes support social interventions, effective implementation across all domains may depend on additional caregiver education and coaching, as emphasized by social learning theory and Naturalistic Developmental Behavioral Interventions (NDBI) frameworks (26). This study’s findings broadly concur with international research emphasizing the pivotal role of caregiver attitudes in intervention success. For instance, a previous study (19) reported that parent-mediated interventions led to significant gains in social communication when caregivers held positive attitudes and received adequate training. Similarly, other study (27) identified that socioeconomic factors and caregiver education strongly influenced parental involvement and child developmental outcomes in LMICs. Our results extend these observations by highlighting contextual factors unique to Iraq, including consanguinity and limited access to autism-specific services, which add complexity to the caregiving landscape.

Compared to high-income countries where comprehensive autism support networks exist, the low prevalence of formal educational participation among mothers in Diwaniyah indicates substantial unmet needs. This gap is concerning given that caregiver education and psychosocial support are consistently shown to reduce parental stress and improve intervention fidelity (22). Consequently, strengthening caregiver training infrastructure is imperative to augment home-based intervention effectiveness in this context.

From a clinical and public health perspective, the study underscores the importance of empowering mothers as primary facilitators of their child’s development through tailored education and psychosocial support. The strong association between maternal attitudes and social skill interventions indicates that enhancing positive perceptions can increase caregiver motivation and intervention adherence. Therefore, programs designed to address maternal knowledge, attitudes, and emotional well-being may yield substantial benefits for children with ASD. Furthermore, the partial implementation of attention and language interventions suggests the need for additional caregiver coaching and resource provision, potentially through telehealth platforms or community health workers, which have shown promise in other LMICs (19). Culturally sensitive adaptations, considering local beliefs, stigma, and family structures, will be essential to optimize these interventions. Additionally, while this study focused on maternal administration of home-based interventions, detailed information regarding the specific types of treatments, the training and monitoring provided to mothers, and the involvement of fathers, siblings, or extended family members was limited. Including such details in future research could provide a more comprehensive understanding of how family dynamics and support influence the effectiveness of home-based interventions.

Finally, a more detailed consideration of sociocultural factors specific to Iraq could further contextualize the findings. Societal beliefs about autism, traditional family structures, prevailing gender roles, and the high prevalence of consanguineous marriage may shape maternal attitudes, caregiving behaviors, and the uptake of home-based interventions. For example, extended family involvement and maternal responsibility norms may both facilitate and constrain engagement in therapeutic activities. Understanding these cultural dimensions is critical for designing interventions that are socially acceptable and feasible. Moreover, the study’s findings have important policy and practical implications: they highlight the need for culturally tailored caregiver training programs, the promotion of community-based support networks, and accessible educational resources to enhance intervention effectiveness in low-resource settings like Diwaniyah. Addressing these factors can help optimize both maternal participation and child developmental outcomes while respecting local sociocultural contexts.

### Strengths and limitations

This study possesses several notable strengths that enhance the credibility and relevance of its findings. The use of census sampling, which included all eligible mothers from primary autism centers in Diwaniyah, ensured a comprehensive and representative sample within the local context. Additionally, the employment of a rigorously developed and culturally adapted questionnaire, validated through expert review and pilot testing, contributed to the reliability and validity of the collected data. Data collection via face-to-face interviews conducted by trained researchers further minimized bias and improved clarity. Importantly, the study’s comprehensive approach, examining multiple developmental domains (social, attention, language) alongside maternal attitudes and intervention practices, provides a holistic understanding of caregiving dynamics in children with ASD.

Several limitations should be acknowledged in interpreting the findings of this study. First, the cross-sectional design limits the ability to establish causal relationships or determine the directionality between maternal attitudes and the use of home-based interventions. Second, the reliance on maternal self-report data may introduce social desirability and recall biases, potentially resulting in overly positive responses. Third, the exclusion of children with severe comorbid disabilities restricts the generalizability of the results to children with less complex ASD profiles. Fourth, the study focused solely on mothers, thereby overlooking the potential role of fathers and other caregivers, whose involvement may significantly influence intervention outcomes. Fifth, the predominantly urban and well-educated sample may have contributed to a positive bias, as these participants might be more familiar with or engaged in the types of interventions assessed. Sixth, the findings are context-specific to Diwaniyah, and caution is warranted when attempting to generalize them to other regions of Iraq or countries with differing sociocultural and healthcare contexts. Finally, home-based intervention implementation was defined broadly as maternal engagement in educational and therapeutic activities at home without specifying frequency, duration, consistency, or diversity. As such, while the study captures overall maternal participation, it does not provide detailed insight into how variations in these dimensions may affect child outcomes, highlighting the need for future research with more nuanced measurement approaches.

## Conclusion

In conclusion, this study provides compelling evidence that maternal attitudes are strongly linked to the use of home-based interventions targeting social skills in children with ASD in Diwaniyah, Iraq. Positive maternal perceptions and higher education levels are associated with greater engagement in supportive caregiving practices, highlighting critical targets for intervention. Addressing gaps in caregiver training and psychosocial support through culturally tailored programs could enhance the effectiveness and sustainability of home-based strategies in this and similar low-resource settings. Future longitudinal and intervention studies are recommended to better understand causal pathways and optimize caregiver-mediated developmental support for children with ASD.

## Data Availability

The raw data supporting the conclusions of this article will be made available by the authors, without undue reservation.
